# HOW BABY BOOMERS SHARE PERSONAL INFORMATION ON PSEUDONYMOUS PLATFORM: AN EXPLORATORY STUDY ON REDDIT

**DOI:** 10.1093/geroni/igae098.2560

**Published:** 2024-12-31

**Authors:** Ern Chern Khor, Moon Choi

**Affiliations:** Korea Advanced Institute of Science and Technology (KAIST), Daejeon, Republic of Korea; Korea Advanced Institute of Science and Technology (KAIST), Daejeon, Republic of Korea

## Abstract

Social media platforms serve as hubs for discussions across diverse topics, fostering connections and information exchange. Pseudonymous platforms allow users to create separate digital identities and facilitating open expression of thoughts and opinions. Previous research tends to focus on younger age groups, resulting in a knowledge gap about older cohorts. This study aims to explore how baby boomers share their personal information on pseudonymous online platforms, using data from Reddit, a popular pseudonymous online platform known for topic-specific communities called subreddits. Data was collected from posts in five subreddits in February 2024, using user flairs (i.e., users’ self-tags of their age range). The dataset encompasses 619 baby boomers and 1.91 million sentences tokenized from generated contents, including both posts and comments. Following Geetha et al. (2021), private information was categorized into three domains: (a) personal relationships, (b) professional-related, and (c) health-related. Following manual annotation, natural language processing was applied to categorize sentences into different domains. Results showed that baby boomers shared personal relationships-related information more frequently than professional-related and health-related information. Compared to younger age groups, baby boomers were less likely to share personal information; however, content analysis revealed age-related uniqueness within the same domain. These findings imply that pseudonymous online platforms could serve as a resource for the aging population seeking information and support on personal relationships issues. However, considering the increased cybercrime risks associated with sharing personal information, future research needs to investigate how to help baby boomers navigate these platforms safely.

